# N-Acetyl-seryl-aspartyl-lysyl-proline Alleviates Renal Fibrosis Induced by Unilateral Ureteric Obstruction in BALB/C Mice

**DOI:** 10.1155/2015/283123

**Published:** 2015-10-05

**Authors:** Gary C. W. Chan, Wai Han Yiu, Hao Jia Wu, Dickson W. L. Wong, Miao Lin, Xiao Ru Huang, Hui Yao Lan, Sydney C. W. Tang

**Affiliations:** ^1^Division of Nephrology, Department of Medicine, The University of Hong Kong, Hong Kong; ^2^Department of Medicine and Therapeutics and Li Ka Shing Institute of Health Sciences, The Chinese University of Hong Kong, Hong Kong

## Abstract

To expand the armamentarium of treatment for chronic kidney disease (CKD), we explored the utility of boosting endogenously synthesized N-acetyl-seryl-aspartyl-lysyl-proline (Ac-SDKP), which is augmented by inhibition of the angiotensin converting enzyme. Male BALB/c mice underwent unilateral ureteral ligation (UUO) or sham operation and received exogenously administered Ac-SDKP delivered via a subcutaneous osmotic minipump or Captopril treatment by oral gavage. Seven days after UUO, there were significant reductions in the expression of both collagen 1 and collagen 3 in kidneys treated with Ac-SDKP or Captopril, and there was a trend towards reductions in collagen IV, *α*-SMA, and MCP-1 versus control. However, no significant attenuation of interstitial injury or macrophage infiltration was observed. These findings are in contrary to observations in other models and underscore the fact that a longer treatment time frame may be required to yield anti-inflammatory effects in BALB/c mice treated with Ac-SDKP compared to untreated mice. Finding an effective treatment regimen for CKD requires fine-tuning of pharmacologic protocols.

## 1. Introduction

Progression of chronic kidney disease (CKD) to end-stage renal disease (ESRD) is characterized by pathogenic mechanisms that converge upon a common pathway leading to progressive interstitial fibrosis, peritubular capillary loss, and destruction of functioning nephrons [1]. Currently, renin-angiotensin system (RAS) blockade with angiotensin-converting enzyme (ACE) inhibitors or angiotensin II receptor blockers (ARB) is currently the best-documented treatment strategy to delay the progression of CKD. However, it remains evident that CKD continues to progress relentlessly to ESRD [2] despite maximal RAS inhibition coupled with stringent blood pressure and glycemic control. The search for a novel therapeutic agent to retard CKD has turned to N-acetyl-seryl-aspartyl-lysyl-proline (Ac-SDKP).

Ac-SDKP is an endogenous tetrapeptide that regulates the proliferation of pluripotent hematopoietic stem cells [3]. It is normally present in the circulation [4] and is exclusively degraded by ACE. There are data to suggest that, in addition to its antiproliferative effects on the hematopoietic system, Ac-SDKP may possess cardiac and renal anti-inflammatory and antifibrotic properties as demonstrated in rat models of hypertension [5, 6], acute myocardial infarction [7], and acute anti-GBM nephritis [8]. Furthermore, studies have elucidated that the renoprotective action of ACE inhibitors may be mediated via Ac-SDKP [9]. However,* in vivo* studies to dissect the renoprotective effects of Ac-SDKP in robust models that recapitulate the pathological changes of human CKD are lacking.

The evolution of CKD to ESRD is well reproduced by unilateral ureteric obstruction (UUO) murine model. Characteristically, macrophage recruitment begins by day 3. In addition, accelerated tubulointerstitial fibrosis with increased matrix deposition and resultant tubular atrophy occur within a week after ureteric ligation [10]. These lesions are highly reproducible in various murine strains including BALB/C mice [11]. In this study, we explored the potential renoprotective effects of Ac-SDKP in the UUO murine model.

## 2. Materials and Methods

### 2.1. Mouse Model of Unilateral Ureteral Obstruction

All animal experiments were approved by the Committee on the Use of Live Animals in Teaching and Research of The University of Hong Kong and were conducted in accordance with the National Institutes of Health Guide for the Care and Use of Laboratory Animals.

Male BALB/C mice (Laboratory Animal Unit, The University of Hong Kong, HK) underwent a sham operation or unilateral ureteral obstruction (UUO) at 12 weeks of age, as previously described [5, 12]. Briefly, mice were subjected to general anesthesia during which the left ureter was exposed through a midabdominal incision and ligated. Sham-operated mice had their ureters exposed and manipulated without ligation. UUO mice were then subjected to the following treatment: (i) vehicle (control), (ii) Ac-SDPK, and (iii) Captopril. Ac-SDKP was delivered at 1 mg/kg/day via surgically implanted subcutaneous osmotic minipumps (Alzet, Cupertino, CA, USA). Captopril was fed by oral gavage at 30 mg/kg/day. All mice were sacrificed on day 7 and the left kidney of all animals was harvested for further analysis. Harvested kidneys were either embedded in 10% neutral buffered formalin for histological examination or snap-frozen in liquid nitrogen and stored at −80°C for further proteomic analyses. Prior to sacrifice, blood samples were collected for the measurement of plasma Ac-SDKP level by enzyme immunoassay kit (Bertin Pharm, France).

### 2.2. RNA Extraction and Real-Time PCR

Total RNAs were isolated from the cortex of the kidney using NucleoSpin RNA/protein kit (Macherey-Negel, Duren, Germany). The mRNA expression was analyzed by ABI 7500 Real-Time PCR System (Applied Biosystems, Carlsbad, CA, USA) according to manufacturer's instructions. Briefly, two micrograms of total RNAs were reverse transcribed to cDNA and subsequently amplified using SYBR Green Master Mix with following primers: ICAM-1, forward-TGGCCTGGGGGATGCACACT and reverse-GGCTGTAGGTGGGTCCGGG; MCP-1, forward-CTCTTCCTCCACCACCAT and reverse-CTCTCCAGCCTACTCATTG; TGF-*β*, forward-AGGGCTACCATGCCAACTTCT and reverse-CCGGGTTGTGTTGGTTGTACA; Col1, forward-TGTGTGCGATGACGTGCAAT and reverse-GGGTCCCTCGACTCCTACA; Col3, forward-ACGTAGATGAATTGGGATGCAG and reverse-GGGTTGGGCAGTCTAGTG; Col4, forward-CCGGGATTTACTGGACCACC and reverse-CCCTTGCTCTCCCTTGTCA; *α*-SMA, forward-GTGCTATGTCGCTCTGGACTTTGA and reverse-ATGAAAGATGGCTGGAAGAGGGTC. Data were analyzed using SDS software (Applied Biosystems). The mRNA expression was normalized with *β*-actin and presented as relative fold change against control mice.

### 2.3. Renal Histology

Paraffin-embedded renal sections (4 *μ*m) were dewaxed using standard sequential techniques and stained with periodic acid-Schiff reagent. Histological changes of the renal cortex were examined under the microscope through a high-power (×400) field. Tubulointerstitial damage was graded from 0–5 (1 ≤ 10%; 2 = 10–25%; 3 = 26–50%; 4 = 51–75%; 6 ≥ 95%) as described previously [13, 14]. Change in renal fibrosis was determined in Masson's Trichrome stained kidney section.

### 2.4. Immunohistochemistry

Immunohistochemical analyses were performed on paraffin-embedded renal sections (4 *μ*m) as previously described [15]. The sections were quenched with 3% hydrogen peroxide, blocked by 2% BSA, and then stained overnight with anti-F4/80 (AbD Serotec, Kidlington, UK), anticollagen 1, and anticollagen 3 antibodies (Southern BioTech, Birmingham, AL, USA). The stained sections were subsequently incubated with peroxidase conjugated secondary antibodies (Dako, Carpinteria, CA, USA). The immunocomplexes were visualized using DAB substrate from Envision Plus system (Dako). All sections were counterstained with hematoxylin before mounting. The number of F4/80^+^ cells in the tubulointerstitium was counted in 10 equivalent high-power (400x) fields and was expressed as the average number of cells per square millimeter.

### 2.5. Western Blot Analysis

Protein from renal cortical tissue was extracted with RIPA lysis buffer (Millipore, Bedford, MA, USA). Equal amount of proteins was resolved in 12% SDS-PAGE gel and transferred to PVDF membrane. After blocking with 5% nonfat milk, the membranes were incubated overnight with anti-*α*-SMA (Sigma-Aldrich, St. Louis, MO, USA) and subsequently incubated with peroxidase conjugated secondary antibodies. The immunocomplex was visualized with ECL prime chemiluminescence (GE Healthcare, Buckinghamshire, UK) using ChemiDoc XRS+ system (Bio-Rad, Hercules, CA, USA). Quantification of protein bands was performed by the ImageJ program (NIH, Bethesda, MD, USA) and was normalized to *β*-actin level.

### 2.6. Statistical Analysis

All data were expressed as mean ± SEM from three independent experiments. The difference between groups was evaluated by unpaired* t*-test using GraphPad Prism, version 5 (GraphPad Software, San Diego, CA, USA). Data were considered statistically significant at ^*∗*^ *P* < 0.05; ^*∗∗*^ *P* < 0.01; ^*∗∗∗*^ *P* < 0.001 versus the corresponding group of sham-operated mice; ^#^ *P* < 0.05; ^##^ *P* < 0.01 versus UUO-vehicle animals in the same operation group.

## 3. Results

### 3.1. Ac-SDKP Levels

**Figure 1:**
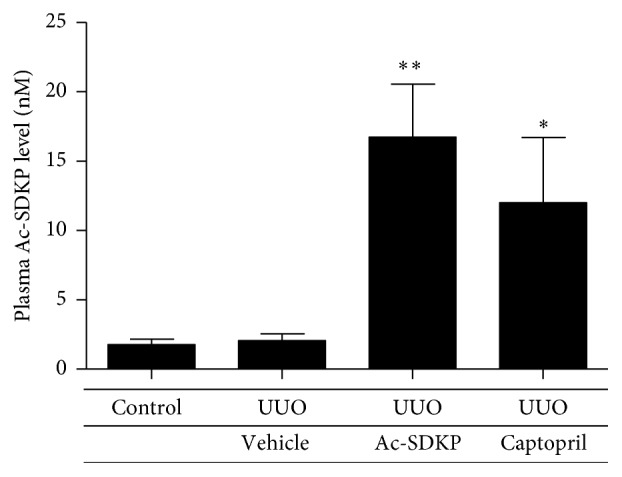
Plasma Ac-SDKP levels in different experimental groups. Data were expressed as mean ± SEM. ^*∗*^ *P* < 0.05; ^*∗∗*^ *P* < 0.01; ^*∗∗∗*^ *P* < 0.001 versus the corresponding group of sham-operated mice; ^#^ *P* < 0.05; ^##^ *P* < 0.01 versus UUO-vehicle animals in the same operation group.

Compared to sham-operated and vehicle mice, Captopril administration significantly elevated endogenous levels of Ac-SDKP in the plasma. Exogenous Ac-SDKP administration was also able to achieve supraphysiological plasma peptide levels (Figure 1).

### 3.2. Morphological Findings

**Figure 2:**
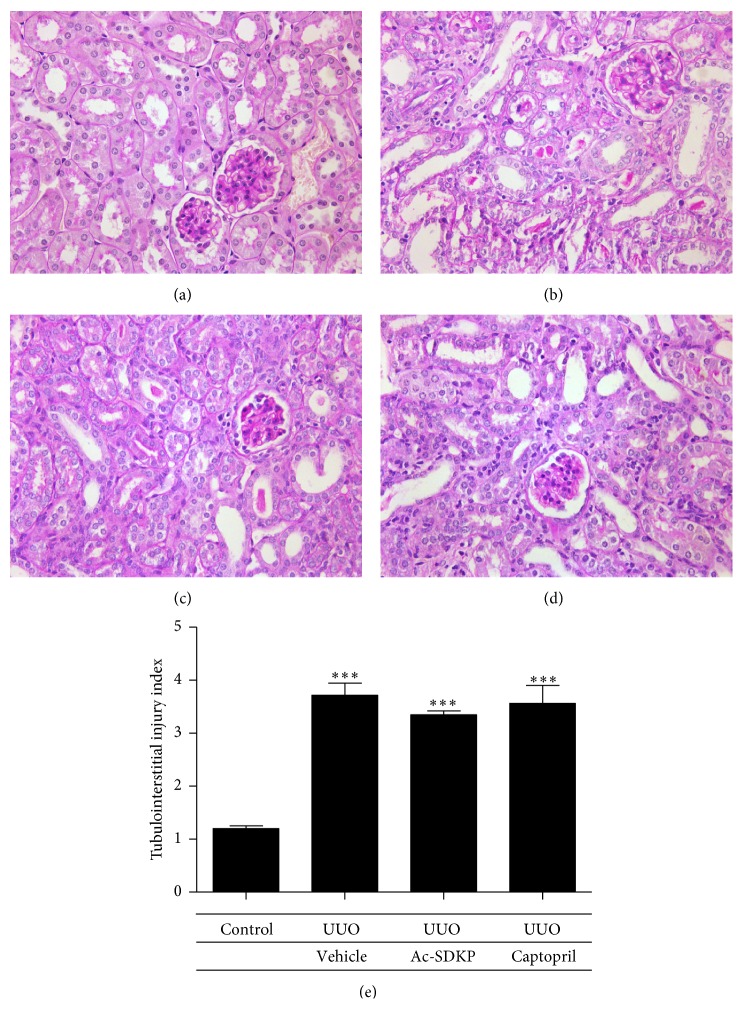
Histological changes. (a–d) PAS staining at day 7 of control animals (a), UUO operated animals (b), UUO animals with Ac-SDKP treatment (c), and UUO animals with Captopril treatment (d). Magnification (400x). (e) Tubulointerstitial injury index of renal cortex for assessing tubular and interstitial damage and graded in an observer-blinded manner from an arbitrary scale of 0–5 (1 ≤ 10%; 2 = 10–25%; 3 = 26–50%; 4 = 51–75%; 5 = 76–95%; 6 ≥ 95%).

Kidneys from the control group as well as the nonobstructed contralateral kidneys from the operated mice groups revealed no histological aberrancies on morphological examination. Kidneys obstructed by ureteric ligation developed severe interstitial fibrosis and tubular atrophy whilst glomeruli were well preserved and unaffected. The level of resultant tubulointerstitial injury compared to the control group was statistically significant. However, following Ac-SDKP or Captopril administration, no significant attenuation of interstitial injury was observed (Figure 2).

### 3.3. Effects of Ac-SDKP on Inflammation

**Figure 3:**
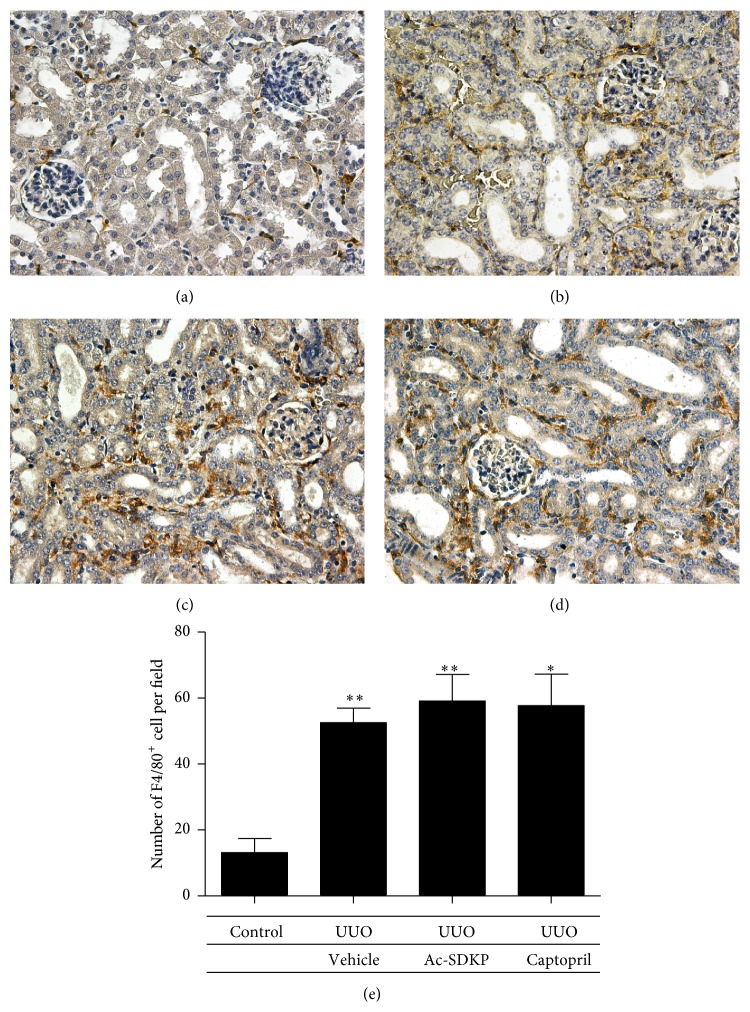
Macrophage infiltration. (a–d) F4/80+ve cells at day 7 of control animals (a), UUO operated animals (b), UUO animals with Ac-SDKP treatment (c), and UUO animals with Captopril treatment (d). Magnification (400x). (e) F4/80+ve cells counted from 10 randomly selected fields.

**Figure 4:**
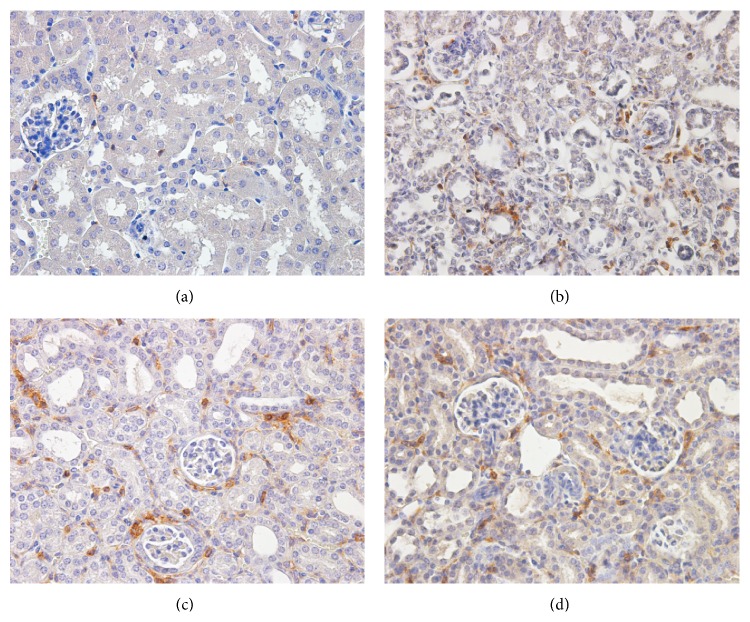
Lymphocytic infiltration. (a–d) CD45+ve cells at day 7 of control animals (a), UUO operated animals (b), UUO animals with Ac-SDKP treatment (c), and UUO animals with Captopril treatment (d). Magnification (400x).

Macrophage infiltration was significantly increased in obstructed kidneys as compared to control kidneys. However, no significant reduction was mediated via the administration of Ac-SDKP or Captopril (Figure 3). Staining for lymphocytes also demonstrated an increase in infiltration following UUO. Similarly, no observable decrement was produced after the administration of Ac-SDKP or Captopril (Figure 4). In concert with this, induced ICAM-1 (an important chemotactic cytokine that mediates cellular adhesion to allow leukocytic transmigration for regions of inflammation) was not downregulated by Ac-SDKP or Captopril (Figure 9).

### 3.4. Effects of Ac-SDKP on Fibrosis

**Figure 5:**
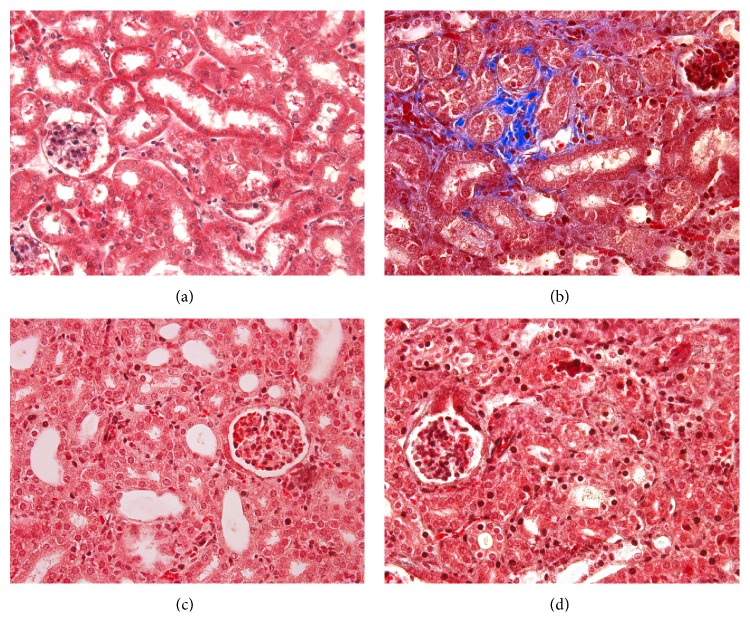
Renal fibrosis by Masson's Trichrome staining at day 7 of (a) control, (b) UUO treatment, (c) UUO with Ac-SDKP treatment, and (d) UUO with Captopril treatment group. Magnification (400x).

**Figure 6:**
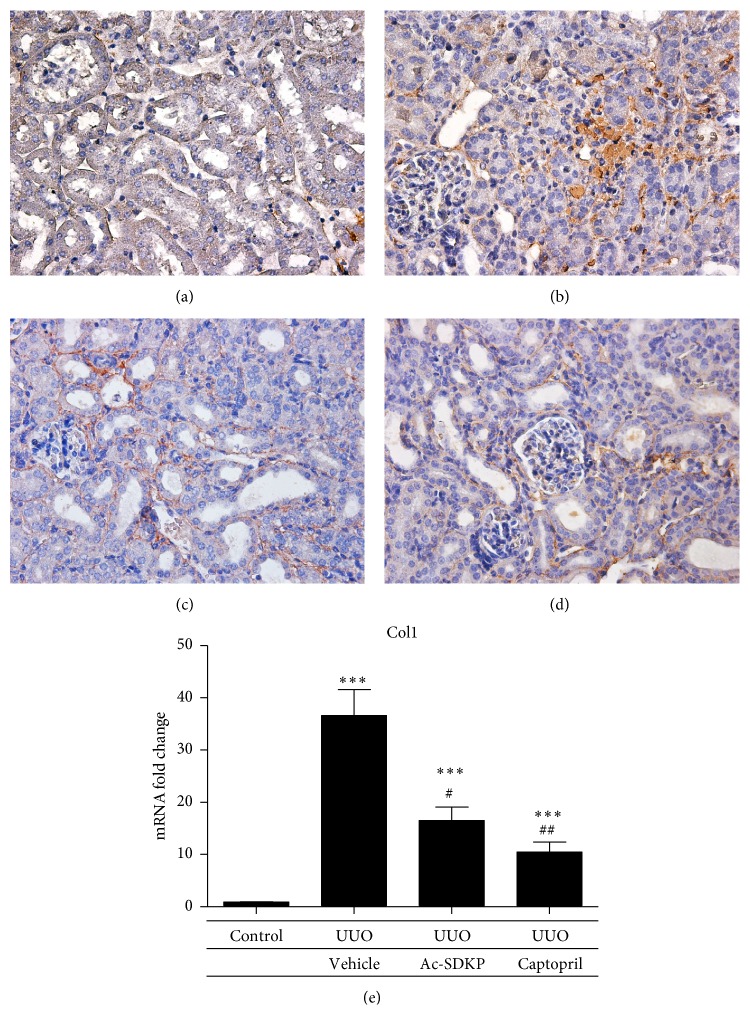
Collagen I expression in renal cortical tissue. (a–d) Representative immunohistochemical staining of protein expression from control animals (a), UUO operated animals (b), UUO animals with Ac-SDKP treatment (c), and UUO animals with Captopril treatment (d). Magnification (400x). (e) Quantitative analysis of mRNA expression by qPCR.

**Figure 7:**
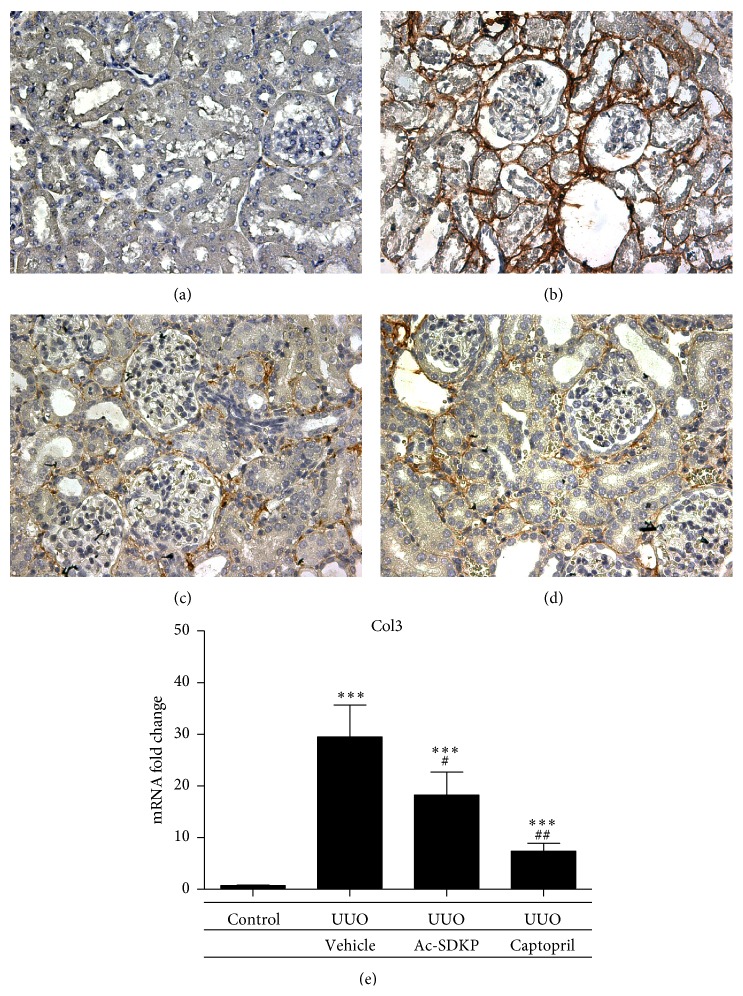
Collagen III expression in renal cortical tissue. (a–d) Representative immunohistochemical staining of protein expression from control animals (a), UUO operated animals (b), UUO animals with Ac-SDKP treatment (c), and UUO animals with Captopril treatment (d). Magnification (400x). (e) Quantitative analysis of mRNA expression by qPCR.

**Figure 8:**
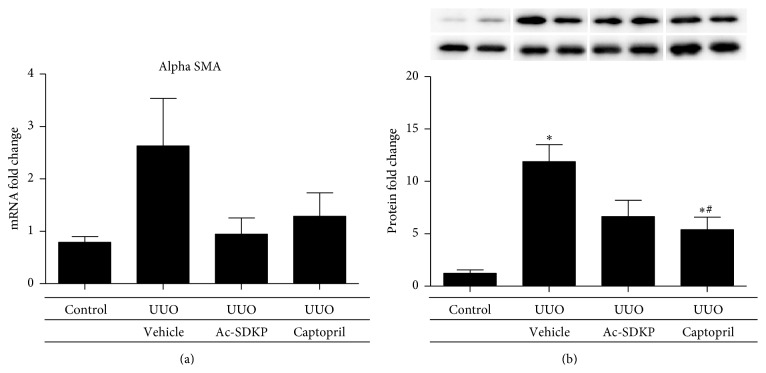
Alpha smooth muscle actin expression in renal cortical tissue. (a) Quantitative analysis of mRNA expression by qPCR. (b) Quantitative analysis of protein expression of different groups, with representative Western blot gels shown in duplicate at the top.

**Figure 9:**
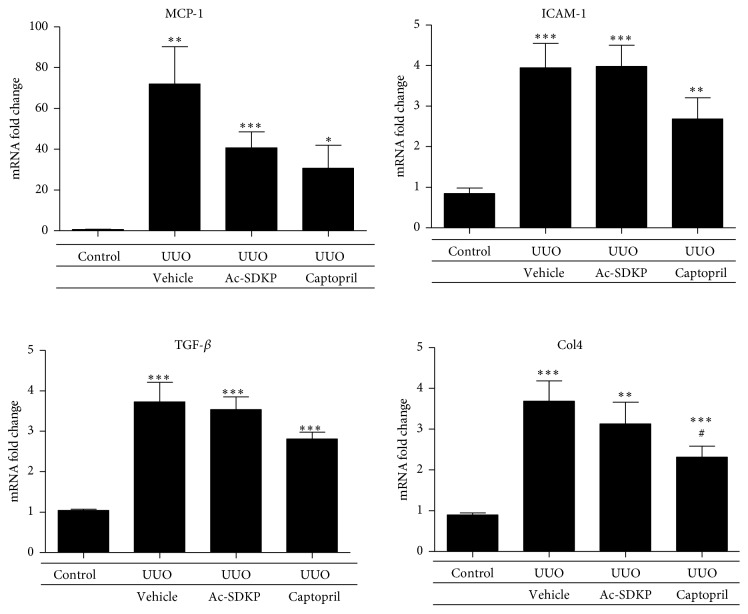
Quantitative analysis of renal cortical mRNA expression of different proinflammatory/profibrotic cytokine genes by real-time PCR in the various experimental groups.

UUO injury induced interstitial expression of collagen fibrils as demonstrated by Masson's Trichrome staining (Figure 5). The expressions of collagen I and collagen III in the renal cortical tissue were both significantly increased by UUO as compared to control. These were both attenuated by the administration of Ac-SDKP and Captopril. Significant reductions in mRNA expression by real-time PCR were demonstrated that was supported by decreased immunohistochemical staining of both collagen 1 and collagen 3 in kidneys treated with Ac-SDKP and Captopril (Figures 6 and 7). A trend towards reductions in collagen IV, *α*-SMA, TGF-*β*, and MCP-1 expression was also observed in the groups treated with Ac-SDKP and Captopril (Figures 8 and 9).

## 4. Discussion

Unilateral ureteric obstruction (UUO) is a robust model that leads to renal injury characterized by tubular cell injury, interstitial inflammation, and fibrosis [16]. Mechanistically, local expression of chemokines following UUO attracts blood-derived macrophages into the renal cortical interstitium. In turn, the cytokines and growth factors secreted by the inflammatory infiltrate result in the accumulation activated *α*-SMA positive fibroblasts in the tubulointerstitial space by a process of recruitment and tubular epithelial-mesenchymal transition (EMT) [10]. The UUO model was therefore a good model for the investigation of the anti-inflammatory and antifibrotic properties of Ac-SDKP. Indeed, when compared to the sham-operated mice, UUO mice developed significant macrophage infiltration and tubulointerstitial fibrosis in the kidney cortex at day 7 after ligation [17].

Previous* in vivo* studies demonstrated Ac-SDKP to significantly attenuate interstitial inflammation and tubulointerstitial fibrosis in deoxycorticosterone acetate-salt hypertensive mice [18] and aldosterone-salt-treated rats [5] and rats induced with acute anti-GBM nephritis [8]. A recent study also found beneficial effects of Ac-SDKP in dampening renal inflammation and fibrosis induced by UUO in Wistar rats [19]. However, in our current study using BALB/C mice, the level of resultant tubulointerstitial injury was not significantly different between the vehicle group and the groups treated with Ac-SDKP and Captopril. The validity of our observations is strengthened by the fact that significant peptide levels of Ac-SDKP were achieved via exogenous administration in the Ac-SDKP group and ACE inhibition in the Captopril group.

One reason that may account for this discrepancy may be the difference in the period of treatment. A shorter treatment duration of 7 days in our study, compared with 14 days in the other study, may not have allowed enough time for the difference in effect to be established. In support of this, UUO induced collagen 1 and collagen 3 gene expression in the cortical tissue were significantly suppressed by Ac-SDKP to suggest an antifibrotic advantage. A similar trend, albeit statistically insignificant, was found in *α*-SMA expression. We therefore postulate that a longer treatment time frame is required to observe a morphological difference in the histology of BALB/C mice treated with Ac-SDKP compared to untreated mice. The expressions of these fibrotic markers were also ameliorated by Captopril, which seems to suggest the actions of Ac-SDKP to parallel that of Captopril or that ACE inhibitors may in part act via raising endogenous levels of Ac-SDKP.

Renal interstitial inflammation which plays a pivotal role in the induction and propagation of interstitial fibrosis is characterized by macrophage and lymphocytic infiltration. In contrast to previous studies, we were unable to demonstrate any anti-inflammatory effects with the administration of Ac-SDKP. Being consistent, this lack of benefit was equally observed in the Captopril group. It is uncertain as to whether this results from a difference in the animal model utilized or that insufficient duration of treatment was employed.

In summary, the UUO model in our study allowed us to demonstrate that Ac-SDKP can attenuate the gene expression of fibrotic markers. The lack of morphological difference detected may be due to insufficient treatment time. Thus, Ac-SDKP may serve as a potential therapeutic agent for the treatment of CKD.
